# Is internal target volume accurate for dose evaluation in lung cancer stereotactic body radiotherapy?

**DOI:** 10.18632/oncotarget.8000

**Published:** 2016-03-09

**Authors:** Jiayuan Peng, Zhen Zhang, Jiazhou Wang, Jiang Xie, Weigang Hu

**Affiliations:** ^1^ Department of radiation oncology, Fudan University Shanghai Cancer Center, Shanghai, China; ^2^ Department of Oncology, Shanghai Medical College, Fudan University, Shanghai, China

**Keywords:** lung cancer, SBRT, ITV, 4DCT, dose evaluation

## Abstract

**Purpose:**

4DCT delineated internal target volume (ITV) was applied to determine the tumor motion and used as planning target in treatment planning in lung cancer stereotactic body radiotherapy (SBRT). This work is to study the accuracy of using ITV to predict the real target dose in lung cancer SBRT.

**Materials and methods:**

Both for phantom and patient cases, the ITV and gross tumor volumes (GTVs) were contoured on the maximum intensity projection (MIP) CT and ten CT phases, respectively. A SBRT plan was designed using ITV as the planning target on average projection (AVG) CT. This plan was copied to each CT phase and the dose distribution was recalculated. The GTV_4D dose was acquired through accumulating the GTV doses over all ten phases and regarded as the real target dose. To analyze the ITV dose error, the ITV dose was compared to the real target dose by endpoints of D_99_, D_95_, D_1_ (doses received by the 99%, 95% and 1% of the target volume), and dose coverage endpoint of V_100_(relative volume receiving at least the prescription dose).

**Results:**

The phantom study shows that the ITV underestimates the real target dose by 9.47%∼19.8% in D_99_, 4.43%∼15.99% in D_95_, and underestimates the dose coverage by 5% in V_100_. The patient cases show that the ITV underestimates the real target dose and dose coverage by 3.8%∼10.7% in D_99_, 4.7%∼7.2% in D_95_, and 3.96%∼6.59% in V_100_ in motion target cases.

**Conclusions:**

Cautions should be taken that ITV is not accurate enough to predict the real target dose in lung cancer SBRT with large tumor motions. Restricting the target motion or reducing the target dose heterogeneity could reduce the ITV dose underestimation effect in lung SBRT.

## INTRODUCTION

With the spiral computed tomography (CT) screening, patients diagnosed with early stage non-small cell lung cancer (NSCLC) were expected to increase significantly in decades [1]. The prognosis for patients with ineligible anatomy lobectomy is disappointed by conventional radiotherapy with a local control rate of 50% [2]. In order to improve the treatment outcomes, stereotactic body radiotherapy (SBRT) with altered dose-fractionation regimens has been investigated and shown promising clinical results compared to the conventional radiotherapy in early stage lung cancer treatment [3, 4].

The lung tumor on conventional CT image can't fully reflect the whole trajectory over the entire respiratory period. The 4DCT technique is capable to obtain the whole tumor motion and tumor deformation over a respiratory period. However, contouring work on 4DCT is tedious and time-consuming. As a result, the target contouring method that delineates gross tumor volume (GTV) over all phases and merge them into an internal GTV (IGTV) is scarcely used. Alternatively, a method delineating an internal target volume (ITV) that encompassing the GTV motion area on maximum intensity projection (MIP) is usually implemented [5, 6]. This ITV or ITV derived planning target volume (PTV) is then used in treatment planning and dose evaluation [7].

This ITV replaces the static GTV for treatment planning and dose evaluation. However, fully including the GTV motion area doesn't mean the ITV is the real GTV or the ITV dose can represent the real GTV dose in dose evaluation. If the tumor size is small [7] or tumor motion is not evident [8], the ITV dose could almost represent the real GTV dose. But the normal tumor size or tumor motion, described in RTOG 0813 trial and the references [9–11], are not always such small. Using advanced intensity modulated radiotherapy (IMRT) the ITV also has comparable dose to the real GTV dose [12]. However, compared to the normal 3D conformal radiotherapy (3DCRT), IMRT is not the first choice and scarcely used in lung SBRT according to the RTOG 0813 trial. So far, there is no literature or work comprehensively and systematically answered whether the ITV dose is accurate to predict the real target dose in lung SBRT in normal tumor motion or tumor size cases.

In this study, we study the accuracy of using ITV for dose evaluation in lung SBRT patients with a wide range of tumor motion and tumor size cases. For this purpose, we designed a series of lung motion phantoms with different tumor sizes and motions. Considering the real clinical scenario was more complicated than phantom experiment, clinical patient study was also included in this work for the validation purpose.

## MATERIALS AND METHODS

### Phantom study

#### Respiratory phantom

**Figure 1:**
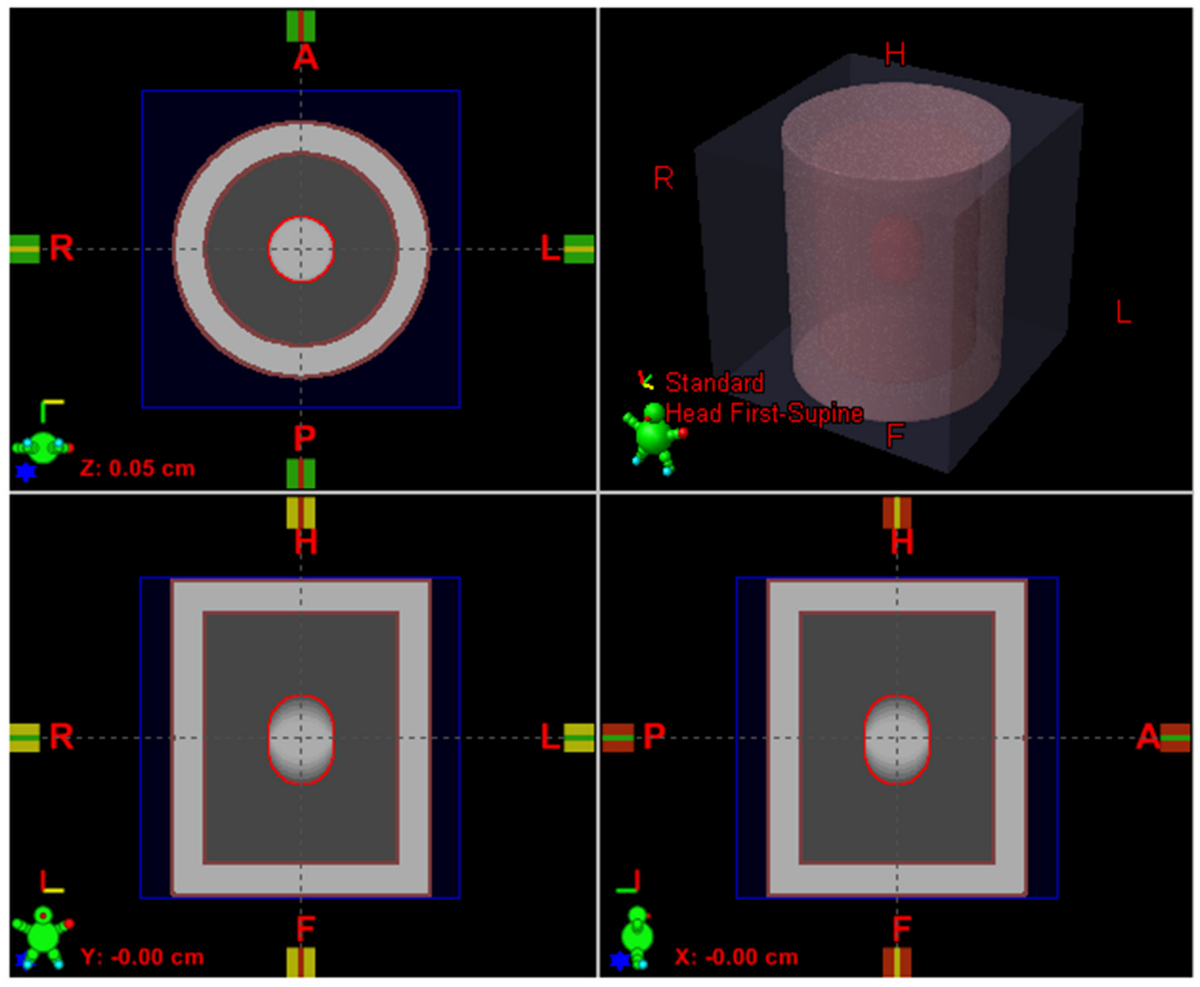
AVG CT of a respiratory phantom case with ITV contoured in MIP (red contour), chestwall (brown contour), and lung (between tumor and chestwall)

Studies have found that the prominent motion happened in the superior-inferior (SI) direction for lung cancer patients and motion amplitude mostly ranges from 0cm to 3cm [9, 10]. The tumor size criteria of patient enroll eligibility in RTOG 0813 trial is within 5cm diameter. Based on reference data above, an in-house program was developed to generate a series of lung cancer phantoms in 4DCT modality that simulated tumor sizes with 1, 2, 3, 4, and 5 cm and rigid motions with 0.5, 1, 2, and 3cm. For each tumor size vs. motion amplitude case, ten breathing phases CT data according to the different motion amplitude was generated. The 0% phase represented the end-of-exhale (EOE: tumor target in peak position) and 50% phase represented the end-of-inhale (EOI: tumor target in valley position). For each breathing phase, the CT values of chest, lung, and tumor were 0, −720, and 0 HU, respectively. These ten phases were used to generate a MIP CT and an AVG CT by maximizing and averaging the voxel intensities over all ten phases, respectively. For example, an AVG CT of lung phantom was shown in Figure 1.

#### GTV and ITV target contouring

GTVs were contoured on all ten phases for each combination of tumor size and motion case. The GTV_x meant the GTV on the 4DCT images with x% phase (CT_x), *i.e.* GTV_20 was the GTV contoured on the CT_20 image (the 20% phase CT image). The ITV was contoured on the MIP images. These targets were auto-delineated using the CT ranger tool (CT range −700, 0) in the Eclipse treatment planning system (TPS, version 8.6.17, Varian Medical Systems, Palo Alto, CA), which was same to manual delineation using the CT pulmonary window as adopted in most NSCLC clinical trials (RTOG 0813 or/and JCOG 0403 trials). The ITV was propagated to the AVG CT for treatment planning and dose calculation as shown in Figure 1. No PTV margin was expanded for simplification purpose.

#### SBRT planning

Treatment plans were designed on the AVG image dataset in the Eclipse TPS using three-dimensional conformal radiation therapy (3DCRT) without plan optimization. The volume dose distribution was calculated using the anisotropic analytical algorithm (AAA) [13] with heterogeneity correction on. Each plan used nine coplanar beams equally for achieving highly conformal dose distribution. The field aperture margin to the target ITV was 5mm. The dose prescription was 50Gy to 95% of the ITV.

The treatment plan on AVG image was then copied to each CT phase and the dose distributions per phase were recalculated with the total number of MUs from the plan divided equally over the 10 phases. Since the phantoms we made without deformation, the GTVs in each CT phase were rigidly registered. After that, the GTV doses per phase were accumulated to create the full GTV_4D dose. The GTV_4D dose was considered as the real target dose over the whole breathing cycle.

The ITV dose based on the AVG image was compared to the real target dose to determine whether the ITV had good dose agreement to real target (GTV_4D). The endpoints for comparison were D_99_, D_95_, D_1_ (doses received by the 99%, 95%, and 1% of the target volume), and V_100_ (relative volume receiving at least the prescription dose). A total of 20 cases with different combinations of target size and target motion were investigated.

### Patient study

#### Patient data

Five patients were enrolled in this study for validating purpose. All 4DCT image data was acquired using Phillips big bore CT simulator (Philips Medical Systems, Cleveland, OH, USA) and retrospectively sorted into ten phases with a slice thickness of 3mm. The ITV was contoured on the 4DCT generated MIP image in CT pulmonary window and copied to the AVG image for treatment planning and dose evaluation. The GTV_0 to GTV_90 targets were also delineated on the CT_0 to CT_90 images. ITV was directly used as planning target without additional margin as the phantom study.

**Figure 2:**
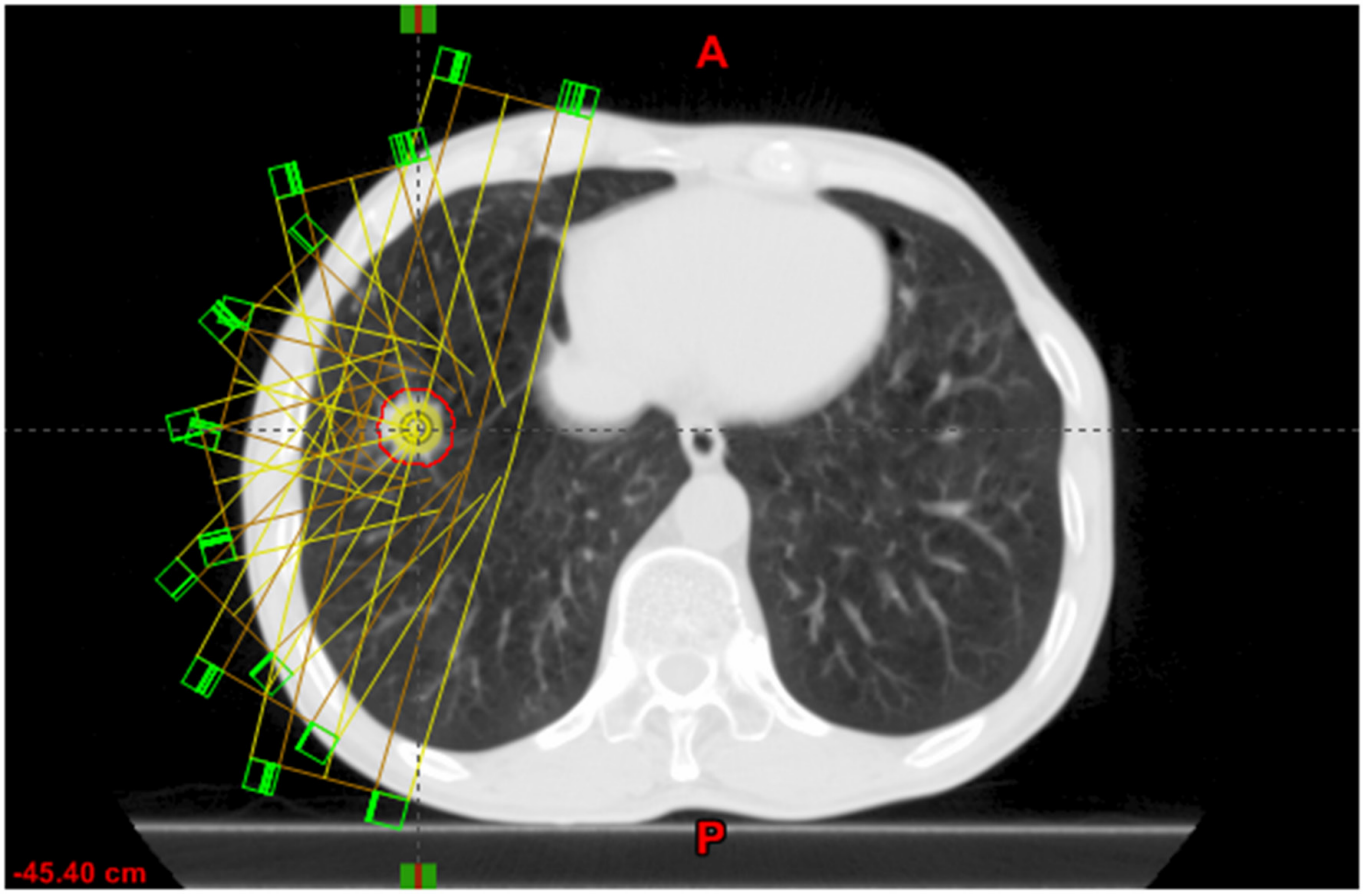
An example of field distribution in SBRT planning in patient case

For each patient, a SBRT plan with 8-10 coplanar fields was designed. Different to the phantom study with equal distributed fields, the fields' distribution in patients study was adapted to the tumor location (see Figure 2). The field aperture margin to the ITV was 5 mm as that in the phantom study. The prescription dose was 50Gy/5 fraction or 50Gy/4 fraction to at least 95% of ITV volume and 90% of the prescription dose covers at least 99% of ITV volume. After creating a SBRT plan on the AVG image, the plan was copied to ten phased CT images with only changing MU of each beam to 1/10 and the dose was recalculated on each 4DCT phase.

The planning data of each phase (CT image, dose and structure) was exported from the eclipse into the Raystation 3.99 (RaySearch Laboratories, Stockholm, Sweden). Each phased CT image was deformed to the CT_30 image using image deformable algorithm [14]. The accuracy of the deformable algorithm was 0.19, 0.28, and 0.17 cm in the LR, AP and IS direction. The full accumulation dose GTV_4D was derived from mapping the dose of other nine phases to CT_30 image phase and aggregating the GTV_30 doses. The GTV_4D dose was considered as the real target dose.

As in the phantom study, the ITV dose was compared to the real target dose through analyzing the endpoints of D_99_, D_95_ and D_1_ and the dose coverage endpoint of V_100_. The lung dose was beyond the purpose of this study and was not analyzed here. All these plans were made only for this study and were not clinically treated on patients.

## RESULTS

### Phantom study

The dose difference of each phantom case was listed in Table 1. For all the checked endpoints, the ITV dose was lower than the real target dose in a range of 0∼8.03% in D_1_, 9.47∼19.80% in D_99_, and 4.43∼15.99% in D_95_ and the ITV dose coverage has a 5% dose coverage loss compared to the real target dose coverage. It meant that the dose evaluation based on ITV would cause to an underdose compared to the real target. For common tumor sizes with 2-5cm and motions of 1-2cm, the actual D_99_ and D_95_ would be 9.81∼19.8% and 5.48∼14.86% higher than those based on ITV; and there was almost no effect on the D1. The ITV dose coverage loss was always 5%, because the V100 of ITV for each phantom case reached 95% and the V100 of the real target reached 100%.

**Table 1:** Dose and target coverage variations between ITV and GTV_4D (real target) in different target size and target motion

Target Size (cm)	Target Motion (cm)			
	0.5	1	2	3
**D_1_ variation (%)**				
1.00	−1.56	−2.87	−8.03	−8.03
2.00	−0.17	−0.08	−1.31	−1.31
3.00	−0.03	−0.08	0.22	0.22
4.00	0.01	0.10	0.10	0.10
5.00	0.00	0.23	0.58	0.58
**D_99_ variation (%)**				
1.00	−16.31	−11.71	−18.05	−17.86
2.00	−11.77	−14.24	−19.80	−19.76
3.00	−12.36	−12.08	−16.14	−15.97
4.00	−10.16	−11.69	−15.72	−14.88
5.00	−9.47	−9.81	−12.65	−12.36
**D_95_ variation (%)**				
1.00	−11.26	−10.52	−15.2	−15.99
2.00	−9.17	−11.42	−14.86	−14.56
3.00	−7.66	−8.87	−11.52	−11.45
4.00	−5.8	−7.12	−9.44	−9.11
5.00	−4.43	−5.48	−7.05	−7.07
**V_100_ variation (%)**				
1.00	−5.00	−5.00	−5.00	−5.00
2.00	−5.00	−5.00	−5.00	−5.00
3.00	−5.00	−5.00	−5.00	−5.00
4.00	−5.00	−5.00	−5.00	−5.00
5.00	−5.00	−5.00	−5.00	−5.00

The dose variation: (D_X_ITV_-D_X_GTV_4D_)/D_X_GTV_4D_ *100%, D_X_ represents D_1_, D_99_, or D_95_. The V_100_ variation: V_100_ITV_-V_100_GTV_4D_.

**Figure 3:**
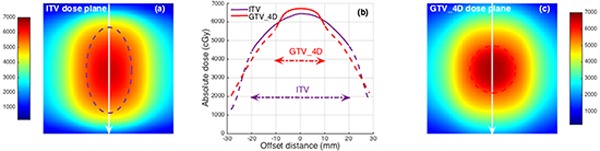
**a.** The ITV target (dash purple ellipse) located on the color washed ITV dose plane. **b.** Dose profile comparison related to ITV and real target (GTV_4D) dose planes in white arrow line position. Dose profiles out of the target were plotted as dash lines. **c.** The real target (dash red circle) located on color washed GTV_4D dose plane.

Figure 3 was a 2-2(2cm *vs*. 2cm in Tumor Size *vs.* Motion Amplitude) phantom case. This case was selected to illustrate the dose deviation between ITV and real target. The ITV (purple dash ellipse in Figure 3a) had a more broad volume than real target (red dash circle in Figure 3c) and contained additional lower dose in the extended volume compared to the real target.

### Patient study

Table 2 listed the tumor characteristic for all five lung patients. Patients 1, 2 and 5 had tumors located at right or left lower lobe. The tumor motions of them were 1.2 cm, 0.7cm and 0.9cm in SI direction, respectively. Patients 3 had a tumor located in left upper lobe and close to the apex. Patient 4 had a tumor located in right upper lobe and adjoined to the thoracic wall. There was no obvious tumor motion observed in Patients 3 and 4.

**Table 2:** Tumor characteristic, dose, and dose coverage analysis of five patients

Patient No	location	size (cm)	Amplitude (cm)	regimine	D_99_(cGy)		D_95_(cGy)		D_1_(cGy)		V_100_(%)	
					ITV	GTV_4D	ITV	GTV_4D	ITV	GTV_4D	ITV	GTV_4D
1	RL	2.5	1.2	50Gy/5f	4694(89.3%)	5258	5048(92.8%)	5440	6665(100.1%)	6657	95.88%(−3.96%)	99.84%
2	RL	1.8	0.7	50Gy/4f	4795(93.7%)	5115	4980(95.3%)	5223	5870(100.5%)	5842	93.75%(−6.12%)	99.87%
3	LU	2.9	0.1	50Gy/4f	4738(98.8%)	4794	4936(99.3%)	4972	5950(100.0%)	5948	93.36%(−0.87%)	94.23%
4	RU	2.3	0.1	50Gy/5f	4787(99.4%)	4814	4949(99.2%)	4991	5604(100.0%)	5601	93.00%(−1.00)	94.00%
5	LL	1.8	0.9	50Gy/4f	4846(96.2%)	5037	4920(94.6%)	5200	6343(99.9%)	6348	92.99%(−6.59%)	99.58%

For location column, RL is right left lobe, LU is left upper lobe, RU is right upper lobe, and LL is left lower lobe.

For D_99_, D_95_, and D_1_ in ITV column, it both listed the absolute dose and the relative dose (in the brackets). The relative dose was the absolute dose of ITV relative to the absolute dose of GTV_4D (real target). For V_100_ in ITV column, the brackets listed the V_100_ difference (V_100_ITV_-V_100_GTV_4D_).

The target doses of D_99_, D_95_ and D_1_ were listed in Table 2. For Patient 1 with a tumor motion of 1.2 cm, the ITV showed an underdose of 10.7% in D_99_ and 7.2% in D_95_, and had a dose coverage loss of 3.96% in V_100_ compared to the real target. Likewise, other two patients (Patient 2 and 5) showed ITV underestimated the real target dose in dose and dose coverage endpoints of D_99_, D_95_, and V_100_. However, the D1 dose of ITV was almost the same to the real target in these three patients. For Patients 3 and 4 with almost no target motion, ITV predicted the dose as almost the same to the real target.

**Figure 4:**
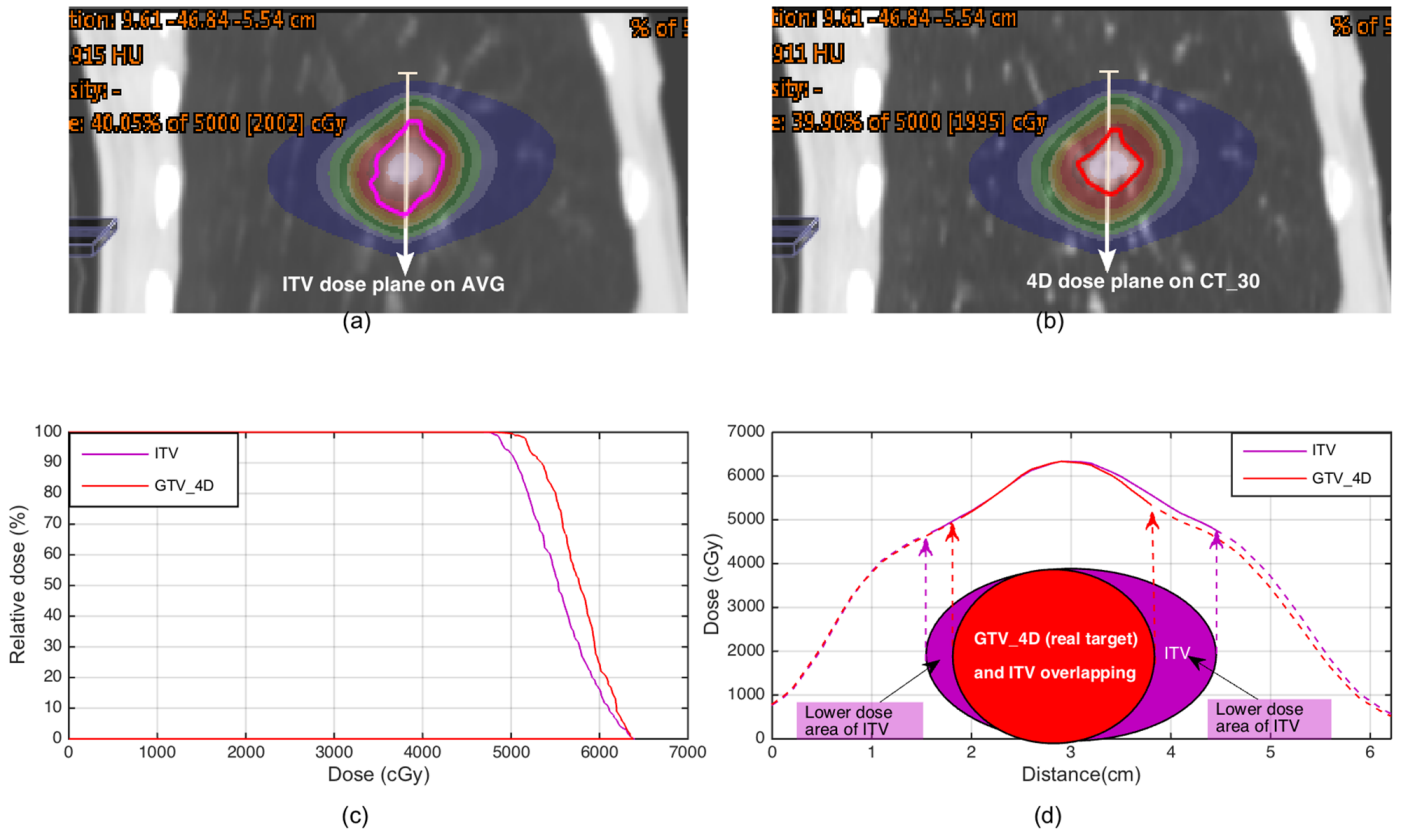
**a.** The ITV target (purple contour) located on color washed dose distribution on AVG CT. **b.** Real target (red contour) located on color washed dose distribution of cumulated plan on CT_30. **c.** Dose volume histogram comparison between ITV and real target. **d.** Dose profile comparison related to the white line position in (a) and (b). The target area and location diagrams of ITV and real target were plotted under the dose profiles. Dose profiles out of the target were plotted as dash lines.

The dose plane and target volume difference between ITV and real target was displayed in Figure 4 for Patient 5. It was clear that the ITV had an additional volume that contained lower dose over the real target (Figure 4d). This caused the ITV had lower D_99_, D_95_, and V_100_ but similar D_1_ compared to the real target as the dose volume histogram (DVH) shown (Figure 4c).

## DISCUSSION

The 4DCT generated ITV has been widely adopted in motion target planning and dose evaluation [7]. However, our study shows it is improper to overuse ITV to represent the real target in dose evaluation.

Compared the ITV dose to the real target dose, the phantom study showed that the D_99_ dose variation ranged from −9.47% to −19.80%, and the D_95_ dose varied from −4.43% to −15.99%. The dose coverage of ITV had a 5% loss in V_100_. It implied that if we used the ITV to evaluate the target dose, we would underestimate the real target dose. Three patients with motions of 1.2, 0.7 and 0.9cm in the SI direction showed that the ITV underestimated the real target dose of 10.7%, 6.3% and 3.8% in D_99_, of 7.2%, 4.7% and 5.4% in D_95_, and the ITV had 3.96%, 6.12%, and 6.59% dose coverage loss in V_100_, respectively. In patients study, the planning technique with different field direction spacings and complicated deformable image registration were used (in Figure 2). But the dose underestimation effects of the ITV remained, which validated the phantom study results.

Clearly, it is easy to see the ITV is broader than the real target volume because of the tumor motion, as shown in Figure 3 and 4. Contrary to the conventional radiotherapy, both RTOG 0813 trial and TG101 report allowed a large target dose inhomogeneity in lung SBRT for hotspots within the central region of a tumor might offer a special advantage in eradicating radio-resistant hypoxic cells that might be more likely located there [15]. Figure 3b and 4d clearly illustrates this dose inhomogeneity in ITV and GTV_4D (real target), which looks like a peak dose distribution. The peak dose distribution resulted in the ITV target contained lower dose in its additional extended volume compared to the real target. This is the reason why the ITV underestimates the real target dose.

Based on the above analysis of the ITV dose underestimation, there are two approaches can be implemented to reduce this effect. One is to reduce the volume variation between the ITV and the real target by respiratory control techniques such as breathe hold [16–18] or gating [19]. By Restricting the motion in a relative small range, the ITV dose underestimation effect would decrease as the Patients 3 and 4 shown in Table 2. The previous study validated that the ITV had the comparable dose to the GTV in very small motion targets (mean target motion of 4.4mm) [8]. Another cause of the ITV dose underestimation effect is the peak dose distribution in the target as mentioned above. For institutions without the respiratory control techniques, reducing the dose heterogeneity in the target is an alternative method to reduce the ITV dose underestimation effect. A previous study [7] reported the biologic effective dose (BED) of ITV was good approximation of the BED of GTV_4D in a mean ITV dose homogeneity index (HI: D1/D99) of 1.14±0.01 in small volume lesions (0.6-3cm^3^). The controversy was that this dose homogeneity biased toward the dose distribution in the conventional radiotherapy (target dose range from 95∼107% (HI: 107/95=1.13) by the ICRU report 50), not in SBRT. This flat dose distribution of target also could not benefit from the advantage of the peak dose distribution in eradicating radio-resistant hypoxic cells that might be more likely located in the central region [15]. The oncologist and physicist should balance the trade-off between the dose accuracy and the potential benefit of peak dose distribution for the lung SBRT with large tumor motions.

## CONCLUSION

ITV can be used to determine the boundary of the target motion and is useful in field aperture setting during treatment planning. However, both phantom and patient study in our work showed the ITV target would underestimate the real target dose in lung SBRT with large tumor motions. Restricting the target motion or reducing the target dose heterogeneity could reduce the ITV dose underestimation effect in the lung SBRT.
